# Supplementary material for: Are carbon emissions trading and green financial instruments synergistic? -Comprehensive quantitative research based on content analysis
Source: PLoS One. 2024 Mar 7;19(3):e0298601. doi: 10.1371/journal.pone.0298601 (PMC10919601; doi:10.1371/journal.pone.0298601)
Supplement: S1 Data — (ZIP) [file pone.0298601.s001.zip › Data/地级市Z_F工作报告.zip!/─┌├╔╣┼/│α╖σ╩╨/└┤╫╘╣1⁄2╓┌║┼í╛┬φ┐╦╩2╛▌═°í┐_2014.docx]

各位代表：

　　现在，我代表市人民政府，向大会报告工作，请予审议，并请市政协委员和列席会议的同志提出意见。

　　一、2013年工作回顾

　　2013年是本届政府任期的第一年，也是经济下行压力持续加大，全市发展遇到较大困难、经受严峻考验的一年。市政府紧紧依靠全市各族人民，深入贯彻党的十八大、十八届二中、三中全会精神，全面落实自治区8337发展思路和市委六届四次全委会安排部署，从全局和战略高度进一步理清思路、选准定位，抢抓机遇、奋力前行，经济发展稳中有进，社会大局和谐稳定。全市生产总值完成1686.2亿元，增长9.2%；公共财政预算收入91.2亿元，增长22.3%；全社会固定资产投资1578.8亿元，增长18.1%；社会消费品零售总额526.6亿元，增长13.2%；城镇居民人均可支配收入20802元，农牧民人均纯收入8022元，分别增长11.4%和13.3%。

　　一年来，我们沉着应对严峻复杂的经济形势，稳运行、调结构、促转型，产业发展水平进一步提升。工业经济增速回升、企业利润降幅收窄、煤电油运先行指标趋稳向好，规模以上工业增加值增长12.2%，工业用电量、石油销售量、铁路发货量分别增长10.8%、7%、16.7%。出台税费减免、电力补贴、信贷支持、延长优惠政策时限等扶持措施，推动企业在困境中稳定生产、降本增效和转型升级；减免各项税费3亿元，发放电价补贴近2000万元，投入技改资金2644万元。大唐Ⅰ系列13.3亿立方米煤制气项目投入运营并实现向北京供气，林西工业园区晋升为百亿级园区。农牧业1571工程扎实推进，新增高效节水种植面积199万亩，总面积达到563万亩，粮食产量实现五连增，达到105亿斤，我市被评为全国产粮大市；家畜存栏连续8年居自治区首位，达到1963万头只，出栏肉牛137.5万头、肉羊730万只、生猪356万口；蒙都荣获中国驰名商标，敖汉小米成为国家地理标志保护产品，阿鲁科尔沁旗被命名为中国草都。服务业快速发展，社会消费品零售总额增速全年保持自治区首位。占地50平方公里的赤峰商贸物流城建设进展顺利，自治区首家保税物流中心获国家批准，红山物流园区实现销售收入260亿元。喀喇沁旗被评为全国休闲农业和乡村旅游示范县，道须沟、玉龙沙湖晋升为国家4Ａ级旅游景区；全年接待游客910万人次，实现旅游收入145.2亿元，分别增长26.4%和32%。

　　一年来，我们千方百计扩大投资规模，上项目、抓招商、促融资，经济发展后劲进一步增强。建立重大项目协调推进机制，动开工3000万元以上重点项目826个，同比增加134个，完成投资1055亿元，增长25.5%。山金10万吨铅冶炼、蓝天糖业100万吨甜菜加工、大唐温都尔花1万千瓦光伏发电等289个项目竣工投产，形成新的经济增长点。积极面向环渤海、长三角、珠三角、港澳台等地开展招商引资和经贸洽谈活动，成功引进庆华煤基清洁能源、中粮肉食产业链等一批大项目，组织了温商赤峰行、苏商赤峰行等大型招商活动，签订了一批新型产业园区、基地等合作协议，与佛山市缔结为友好城市，与北京、河北、辽宁等地的区域合作更加紧密。加强政银合作、银企对接，签订融资协议超百亿元。新增金融机构12家，全市存、贷款余额分别达到1267.6亿元和860.7亿元，分别增长9.4%和20.9%，贷款增速高于存款增速11.5个百分点。全年引进国内区外资金912.5亿元，增长19.1%。

　　一年来，我们持续加大民生投入，保基本、办实事、促和谐，社会建设和管理进一步加强。全市民生支出260亿元，占公共财政预算支出的74.9%，比上年提高5个百分点，高于自治区平均水平11.3个百分点。城镇新增就业3.1万人，登记失业率控制在3.9%以内。企业退休养老金月均提高168元，城镇居民基本医疗保险最高支付限额、新农合报销封顶线分别提高到10万元和25万元。城乡低保、五保及三无人员供养标准进一步提高，救灾救济、养老服务、孤儿保障、公益慈善等工作得到加强。扶持10.2万贫困人口实现稳定脱贫，解决37.8万人饮水安全问题。各项社会事业得到全面发展。新培育国家级科技企业孵化器1家、自治区产业技术创新联盟3家，蒙药产业技术创新联盟成为国家重点培育联盟。义务教育、高中阶段免费教育经费和重点教育工程资金全部落实，内蒙古交通高级技工学校晋升为技师学院，赤峰农牧学校成为国家级示范校。赤峰学院附属医院新址投入使用，自治区卫生云平台赤峰分中心搭建完成。出生人口性别比下降3.4个点。完成35个苏木乡镇综合文化站建设，新增24处国家级文物保护单位，红山文化遗址、辽代上京城和祖陵遗址列入世界文化遗产预备名单。提前2年完成十二五广播电视村村通任务。自治区第十三届运动会场馆建设、筹备工作有序推进。深入开展大接访行动，解决了一批重点领域信访问题，信访总量下降36%。严厉打击各类违法犯罪活动，人民群众安全感进一步增强。强化安全生产和食品药品安全监管，全年无重特大安全事故发生。支持军队和国防后备力量建设，双拥创建成果进一步巩固。

　　一年来,我们加快推进城镇化进程，重规划、强建管、夯基础，城乡发展面貌进一步改善。完成新一轮城市总体规划修编，中心城区控制区面积扩展到近400平方公里。小新地组团、桥北组团、松北新城开发及老城区棚户区改造取得新进展，中心城市建成区面积103.9平方公里，人口增加到94.8万。友谊大街全线贯通，大板桥、友谊大桥、新华大桥建设进展顺利，昭乌达桥拓宽改造完成。新增供热能力350万平方米，集中供水、燃气普及率分别达到95.2%和96%。新开通公交线路5条，增加新能源公交车200辆。开展治堵保畅专项行动，坚持改造设施与加强管理同步推进，交通拥堵得到有效缓解。全市新开工房地产面积526万平方米，建成各类保障性住房5166套。完成老旧小区改造142万平方米，实施农村牧区危房改造2.9万户。各旗县城镇规划建设管理水平进一步提高，辐射带动能力明显增强。完成生态、交通、水利、电力等基础设施建设投资92.2亿元。新增营造林301万亩，完成重点区域绿化20.1万亩，森林覆盖率提高到35.3%。赤承高速建成通车，我市公路交通全面融入华北路网，大大缩短进京时间。新增通村油路1221公里。赤锦、白浩铁路具备通车能力。新增通航城市5个，进出港旅客达到66万人次，增长73.7%。三座店水库移民工作基本完成。220千伏绍根输变电工程投入运行。严格土地、水及矿产资源管理，资源综合开发利用水平进一步提高。较好地完成了自治区下达的节能减排任务。

　　一年来，我们注重和加强政府自身建设，转作风、提效能、树形象，公共服务水平进一步提高。认真落实中央、自治区和市委关于改进工作作风、密切联系群众的各项规定，修订和完善《市政府工作规则》，精简会议活动和文件简报，改进调查研究和新闻报道，狠抓作风转变和工作落实，政府工作效能进一步提高。市政府会议和发文数量分别减少8.9%和13.5%。深入开展肃纪整风专项活动，纠正和查处干部纪律作风问题631个。全面清理超标办公用房和违规公务用车，严禁新建政府性楼堂馆所，严格控制一般性支出，全市三公经费支出下降35.3%。认真清理政府性债务，加强规范化管理。大力简政放权，取消、下放行政审批事项143项，占全部审批事项的45.4%。市政务服务中心办事大厅由950平方米增加到4200平方米、入驻部门由24家增加到41家。加强和规范公共资源交易，完善财政投资评审制度，节约资金20.8亿元。自觉接受人大依法监督和政协民主监督，广泛听取各民主党派、工商联和无党派人士意见，认真办理人大代表建议83件、政协委员提案177件，办结率100%。积极推进政务公开，加强行政监察、经济责任审计和社会监督。加强政府法制工作，行政执法水平进一步提高。

　　2013年，我们成功承办了中国北方农业科技成果博览会、第五届中国苜蓿发展大会、中国飞来者大会、第七届全国篆刻艺术展、第二届中国印城文化节等国家级会展活动，第二次被评为全国百姓最具幸福感城市，荣获国家森林城市、自治区园林城市称号，进一步提升了赤峰的知名度和影响力。

　　上述成绩的取得，是自治区党委、政府和市委正确领导的结果，是全市上下团结一心、奋力拼搏的结果。在此，我代表市人民政府，向全市各族人民，向在各条战线上辛勤工作、付出努力的同志们，向所有关心和支持政府工作的朋友们，表示崇高的敬意和衷心的感谢！

　　在总结成绩的同时，我们也清醒地认识到，在经济下行压力下，制约发展的一些困难和问题进一步显现。一是经济总量小、结构不合理、产业层次低。冶金产业所占比重大、链条短、抗风险能力弱，装备制造等战略性新兴产业发展慢、贡献率低，服务业和中小企业发展仍显滞后。二是保持经济持续增长动力不足。拉动作用较强的大项目少，项目质量不高、推进不快，一些项目签约未落地、竣工未达产、投产未达效。三是基础设施瓶颈制约严重。能源外送通道建设滞后，水资源保障能力不强，环境容量承载压力较大。四是城镇化水平低。城乡二元结构突出，村庄基础设施滞后，城市交通拥堵、环境污染、空气质量下降等问题逐步显现，城镇承载、集聚和辐射带动能力不强。五是民生领域仍有许多薄弱环节。房价高、看病贵、就业待遇低、优质教育资源短缺、养老服务设施不足等问题仍很突出，城乡居民增收、社会保障、扶贫攻坚、安全生产、信访维稳、平安建设等任务仍很繁重。六是政府自身建设仍需加强。政府职能转变有待进一步加快，四风问题仍不同程度存在，不作为、乱作为、效率低、办事难等现象还时有发生。对这些问题，我们要以强烈的责任感和紧迫感，积极谋求破解之策，下大力气逐步加以解决。

　　二、2014年主要任务

　　今年政府工作的总体要求是：深入贯彻落实党的十八大、十八届二中、三中全会和习近平总书记系列重要讲话精神，深入贯彻落实自治区8337发展思路和市委六届五次全委会议精神，全面深化改革，坚持稳中求进，全力保增长、调结构，进一步提高经济发展的质量和效益，进一步统筹城乡发展，进一步改善民生，进一步促进社会和谐稳定和公平正义，为全面建成小康社会奠定坚实基础。

　　主要预期目标是：地区生产总值增长9%左右，公共财政预算收入增长7.5%，全社会固定资产投资增长16%，规模以上工业增加值增长12%，社会消费品零售总额增长13%，城乡居民收入分别增长10%和11%；城镇登记失业率控制在4%以内，居民消费价格总水平涨幅控制在3.5%左右，完成自治区下达的各项节能减排任务。

　　完成上述目标，有机遇、有条件、有潜力，也有挑战、有难度、有压力。面对依然复杂严峻的宏观经济形势，我们必须牢牢把握发展大局，充分估计可能遇到的困难和问题，既要坚持底线思维、早做预案、科学应对，又要坚定信心、奋发有为、攻坚克难，争取把各项目标任务完成得更好，努力在区域发展中开创新局面、再有新作为。

　　重点要做好以下八个方面的工作：

　　（一）围绕保增长，在增投资、上项目方面有新作为，进一步增强发展后劲

　　突出抓好投资。用好用足本级财政投资，努力争取上级投资,有效增加金融投资，多渠道激活社会投资，全方位开展招商引资。充分发挥政府投资的杠杆作用，采取贷款贴息、融资担保、以奖代补等措施，支持工业园区、支柱产业、重大项目、重点企业、民营经济和中小企业加快发展。准确把握国家和自治区政策导向，积极争取上级项目和资金支持。大力改善金融环境，争取更多股份制银行及证券、投资、基金公司来我市设立分支机构、开展业务，支持小贷公司、担保公司、典当企业等健康发展，引导金融机构扩大融资规模，激活民间存量资金；做好政府专项债券和企业直接融资工作，积极争取国债资金，扎实推进企业上市工作；力争全年贷款余额突破1000亿元。充分利用社会投资，鼓励和引导社会资本踊跃进入经济社会建设领域。围绕重点领域和重点产业持续加大招商引资力度，积极承接发达地区产业转移，加强与周边盟市区域合作，创新京蒙合作机制，主动融入环渤海经济圈；全年引进国内区外资金突破1000亿元。

　　加快重点项目建设。紧紧围绕五大基地建设，组织实施一批具有全局性、基础性、战略性影响的重大项目，抓好列入自治区调度的58个重点项目建设，确保按计划完成投资。推动工业投资加快进度、增加强度，努力保持较快增长势头，着力实施投资1000万元以上重点工业项目，确保年内完成投资655亿元；下大力气解决竣工项目达产达效慢、生产效益不高等问题，切实推动一批已建成项目成为新的经济增长点。加快经锡高速、省道304乌兰布统至海拉苏段、桥北客运站建设，开工建设国道303大板至林西段，确保公路完成投资28亿元。加快巴新、林白铁路及集通增二线建设，开工建设锦白铁路复线，加快推动赤峰至京沈客专连接线前期工作，力争铁路完成投资31亿元。加快三座店水库向中心城区引、供水工程建设，推动东台子水库前期工作取得进展，启动打虎石水库向宁城工业园区供水、西拉沐沦河与老哈河连通、琥珀沟水库等工程前期工作，确保水利工程完成投资13亿元。加快白音诺尔220千伏、阿北220千伏等42个输变电项目建设，确保完成投资12亿元。

　　发展壮大园区经济。坚持把园区打造成县域经济的主阵地、经济增长的主引擎，统筹规划园区布局和项目摆布，进一步突出园区产业发展定位和转型升级目标路径，努力形成特色鲜明、优势互补的园区经济新格局。进一步完善园区优惠政策，加大园区基础设施建设力度，集约利用园区土地、水、电等基本要素资源，完善园区利益分享机制，确保入园企业享受政策更优、运行成本更低、发展环境更好、发展空间和潜力更大。加快红山、元宝山、安庆三个园区相向发展，构建赤峰东部产业带。积极争取将我市列入国家级承接产业转移示范区，推动敖汉、左旗、阿旗三个工业园区晋升自治区级园区，实现每个旗县区都有自治区级工业园区的目标，进一步推动各旗县区进行园区整合和扩区升级。依托资源禀赋和产业基础，加快推进克旗煤化工、右旗煤电化、林西县氟化工、翁旗机械制造、喀旗有色金属加工、红山区农畜产品加工等特色园区建设，构建一批产业集群，增强园区发展后劲和集聚发展能力。

　　（二）围绕五大基地建设，在加快结构调整方面有新作为，进一步提升产业发展层次和水平

　　加快工业转型升级步伐。推进庆华清洁能源、大唐煤制气二期、渤海化工、中海油煤化工等项目前期工作，争取尽早开工建设，确保大唐煤制气一期Ⅱ系工程竣工投产。推进罕山、华能书声等风电基地项目建设，继续推动大唐2×66万千瓦超临界、国网新源120万千瓦抽水蓄能等项目前期工作，确保中电投2×30万千瓦热电联产项目开工建设。推进风险探矿、深部探矿和外围找矿，增强矿产资源的后续保障能力。增加科技研发投入，集中力量攻关一批技术难题，提升重点企业、产业的核心竞争力。加快建设有色金属产业研究院，培育4家国家级高新技术企业，提升我市产业升级和产品创新水平。抓好中色五期14万吨锌冶炼、云铜10万吨铜冶炼扩建等项目前期工作，推动亚鼎铜业、贺麒铜业等加工型企业增加品种、延伸链条。积极引进战略投资者和行业领军企业，通过合并重组、股权转让、资产置换等方式，壮大食品、制药、纺织、建材等传统产业。加强与国内知名大企业大集团战略合作，推进已签约的清华大学新能源汽车产学研基地、中关村海淀园赤峰分园、台湾陶瓷产业园等项目尽快落地实施，抓紧建设浩克新能源汽车、奇瑞农机制造、拓佳液晶显示屏等装备制造业项目。加快云计算孵化园区和数据中心建设，发展云计算应用服务和硬件设备制造，壮大蒙东地区云计算产业基地。

　　大力发展现代农牧业。扎实推进粮食安全工程和节水增粮行动，粮食播种面积稳定在1600万亩以上，产量稳定在100亿斤以上。总结经验，创新机制，抓好玉米膜下滴灌成果巩固，确保完成新增100万亩任务，总面积力争达到400万亩。抓好蔬菜、花卉、食用菌等设施农业，确保总面积突破100万亩。大力发展林草产业，新增节水紫花苜蓿30万亩、节水经济林20万亩。加快发展现代畜牧业，推进牲畜良种化步伐和标准化养殖，完善疫病防控体系和机制，全年出栏生猪400万口、肉牛150万头、肉羊800万只，肉蛋奶产量增长5%以上。大力推进农牧业产业化经营，加强农畜产品营销渠道建设，加快培育家庭农牧场、种养大户、农牧民合作组织及产业化龙头企业，进一步提高农牧业规模化、专业化、标准化、信息化水平。抓好伊品生物10万吨苏氨酸、恒都10万头肉牛、中粮150万口生猪产业链等项目建设，提升农畜产品产业化和精深加工水平。加快实施品牌战略，培育更多的无公害农畜产品、绿色食品、有机产品、地标产品，提升我市农畜产品知名度和竞争力。

　　进一步提升服务业发展层次。注重发展生产性服务业，大力推进赤峰商贸物流城建设，推动金融物流港、保税物流中心尽快建成并投入使用。积极发展第三方物流和冷链物流。吸引国内外知名物流企业来我市建立区域分支机构，带动区域物流业做大做强。全年物流业销售收入突破500亿元。搞活生活性服务业，抓好中心城区和城关镇商圈建设，引进更多知名商贸品牌、新型业态和城市综合体，推动传统服务业提档升级。加快培育年销售额超10亿元的商贸市场，确保万达广场、上海城、东方永业广场等项目开业运营。加快新型基层供销合作组织及经营服务体系建设，扩大新网工程覆盖面。培育发展信息咨询、教育培训、养老服务、体育健身、包装设计等更多消费热点。推进文化旅游融合发展，办好中国飞来者大会、巴林石文化节、美林谷滑雪节、越野e族汽车拉力赛等活动，形成独具特色的四季旅游新亮点。加大旅游宣传推介力度，全方位开展与北京、承德等重点地区的旅游合作，创建更多的无障碍旅游区和旅游线路，力争开通香港等城市旅游包机。全年接待游客突破1100万人次，旅游收入达到180亿元。大力发展古玩、书画、出版、演艺等传统文化产业，加快发展创意、设计、会展、动漫等新兴文化产业，实现增加值54亿元。

　　（三）围绕全面深化改革，在推进重点领域和关键环节的改革方面有新作为，进一步释放改革红利和激发市场活力

　　加快经济领域改革。支持非公有制资本通过出资入股、收购股权等多种方式参与国有企业改制重组及合资经营，推动国有、集体、非公有制资本交叉参股、相互融合。理顺国有资产监督管理体制，着手组建国有资本运营公司或投资公司。在交通、文化、卫生、教育等领域整合相关国有资产，引入社会资本，逐步组建股份制投资公司。支持非公有制经济发展，坚决清除市场壁垒，清理、废止妨碍统一市场和公平竞争的各种规定。推进工商注册登记制度改革。深入推进集体林权配套改革，探索试点国有农牧场乡镇化管理。鼓励民间资本参与地方金融机构增资扩股、设立民营银行，积极申报赤峰商业银行。加大对金融机构监管力度，严厉打击非法集资。基本完成农村牧区集体土地所有权确权登记发证工作，推进宅基地及集体建设用地使用权登记发证工作。启动农牧民住房财产权抵押、担保、转让试点，鼓励土地承包经营权向专业大户、家庭农牧场、农牧民合作社、农牧业企业流转。实行企业投资项目备案制，由企业依法依规自主决策。把政府性债务纳入预算管理，规范举债权限和规模，严格控制新增债务。全面公开财政预决算、部门预决算及三公经费预决算。

　　推进社会领域改革。进一步深化政府机构改革，严格执行机构限额，严格控制编制总数，试点在同一层级内实行人员编制动态管理、余缺调剂；逐步将从事生产经营活动的事业单位转企改制；科学配置机构权利和职能，推行市县乡三级政府及其工作部门权力清单制度。探索政府购买服务，在政府网站建设、大型会展和节庆活动、物业管理、市政保洁、垃圾清运等事务性管理服务方面，引入市场机制，通过合同、委托等方式向社会购买，并逐步推广到由政府承担的基本公共服务领域。推动公办事业单位与主管部门理顺关系，探索建立事业单位法人治理结构，推行文体公益性场馆组建理事会，吸纳有关方面代表、专业人士、各界群众参与管理，提高公益服务能力和水平。整合基层教育、文化、体育、科普等方面的资金、项目、场所、设施、人才等资源，建设综合性文化服务中心，满足群众多样化需求。推进以办学条件、师资队伍、教育质量为重点的城乡义务教育均衡发展，下大力气解决城乡、校际之间差距过大问题；逐步取消设置重点学校重点班，解决择校热难题；试点建立校长公开竞聘、教师定期交流机制，推行学区制和九年一贯对口招生；推进中考招生制度改革，试行初中学业水平考试制度和学生素质评价制度；建立学科设置与企业需求联动配套机制，加快职业教育集团化发展。推进公立医院改革，建立科学的医疗绩效评价机制和适应行业特点的人才培养、人事薪酬制度；取消以药补医，降低群众看病成本。落实好单独两孩生育政策。

　　（四）围绕两型社会建设，在加快转变发展方式方面有新作为，为建设美好家园留住蓝天绿地、碧水青山

　　实现资源集约节约利用。加快矿产资源整合，提高开发门槛，新建矿山企业一律配套建设加工转化项目；按照谁开发、谁保护，谁污染、谁治理原则，规划建设矿山公园，创建和谐矿区、绿色矿区。实行最严格的水资源管理制度，在全社会广泛开展节水行动；强化水源地保护，确保全市水源安全；扩大节水农牧业规模，严禁使用地下水发展高耗水产业，加快中心城区10万吨污水处理、10万吨中水回用工程建设，逐步使符合条件的工业企业全部使用中水和地上水，减轻地下水开采压力。大力推进高标准基本农田建设，切实保护好耕地；加强项目用地管理，开展闲置土地专项清理，杜绝圈而不建、占而不用行为。

　　抓好节能减排。建立和完善节能减排市场化机制，强化节能减排目标责任制和问责制，实行能源消费总量和能耗强度双控考核。强化安全、环保、能耗、土地等约束，严格控制高耗能、高排放和产能过剩行业投资，防止低水平重复建设和盲目扩张，加快淘汰落后产能。强化工业、交通、建筑、公共机构等重点领域节能工作，加快既有建筑节能改造，推广合同能源管理及绿色照明和新型绿色建筑材料应用，在政府投资建设领域强制执行绿色建筑标准。抓好火电厂、钢铁厂、水泥厂、污水处理厂、畜禽养殖场和机动车等重点领域减排，力争实现有色金属企业在线监测全覆盖。

　　实施蓝天行动。严格落实《赤峰市中心城市大气环境综合整治方案（2013－2017）》，持续改善城市空气质量。启动实施PM2.5、一氧化碳和臭氧实时监测工作。加大重点区域和重点污染源的综合整治力度，推动两高项目退出生态脆弱区域。启动蒙欣制药、艾克制药等企业搬迁改造，有序推进中心城区工业企业退城进园。推动克旗至赤峰中心城区及旗县区输气管道建设，鼓励使用清洁能源。加快公交车及出租车油改气进度，切实抓好秸秆禁烧工作，加强建筑工地扬尘、城镇燃煤小锅炉等治理，严控工业废气排放。启动全国环境保护模范城市创建工作。

　　构筑城乡绿色空间。按照总体规划、分步实施原则，启动中心城区一环两线三山五河景观系统提升改造工程，即对环城高速公路用地范围外及沿线山体进行绿化，对市区公路及铁路沿线的景观、建筑物、道路设施和环境卫生进行综合整治，对红山、南山、西山进行生态景观建设，对锡伯河、英金河、阴河、半支箭河、昭苏河城区河道进行综合治理。启动实施锡伯河百公里水利风景观光带工程，建设集水生态文明、观光旅游、休闲度假于一体的特色生态景观长廊。继续实施京津风沙源治理、樟子松基地建设，完成营造林面积100万亩。投资12亿元，完成重点区域绿化面积24万亩。完成水土保持综合治理面积105万亩。加大沙地治理力度，落实好草原保护补助奖励、草畜平衡、封育禁牧等政策措施。巩固国家森林城市成果，争创国家园林城市。

　　（五）围绕经济社会协调发展，在改善民生和为民办实事方面有新作为，使改革发展成果更多地惠及各族群众

　　推进稳定型就业创业。实施更加积极的就业政策，大力发展劳动密集型产业、服务业和非公有制经济、中小微企业，提供更多的就业岗位，确保城镇新增就业2.8万人。加快建设创业型城市，在市场准入、税费减免、信贷融资、基金奖励、创业培训等方面提供支持，建成一批小微企业创业园和创业孵化基地，鼓励支持高校毕业生、复转军人和返乡农民工自主创业。突出抓好城镇困难群体、特殊群体就业工作，动态消除城镇零就业家庭。以保障本地企业用工需求为重点，开展各类人才招聘活动，促进更多的劳动者实现就近就业。打造更多的劳务品牌，切实增加劳务收入。依托全市中高职院校、培训机构，培养更多满足发展需要的技能型人才。深入实施玉龙人才工程，抓好人才引进和培养工作。

　　加强共享型社会保障。继续扩大社会保险覆盖面，稳步提高社会保险统筹层次和标准，落实好提高企业退休人员基本养老金政策，城镇职工和城乡居民养老保险参保人数分别达到49.6万人和162万人，城镇职工和城镇居民医疗保险最高支付限额分别提高到30万元和12万元。城乡低保标准每人每月分别提高15元和12元，五保供养标准在上年基础上提高12%。完善医疗救助、临时救助等社会救助制度，推进社会福利事业发展，积极做好孤儿、残疾人、流浪乞讨人员等弱势群体保障工作。加强老龄服务工作，将养老服务体系建设纳入城乡建设总体规划，推进养老服务设施建设，建成一批城镇老年养护院、社区老年人日间照料中心、社会福利院及乡镇敬老院、农村牧区养老幸福院；落实老年人优待政策。扎实推进扶贫攻坚工程，建立精准扶贫机制，认真落实规划、项目、干部三到村三到户帮扶举措，充分利用金融资本、社会资本参与扶贫开发，确保实现14万人稳定脱贫。

　　发展普惠型社会事业。面向市场、企业和广大农牧民，承办好2014赤峰·中国北方农业科技成果博览会。投入资金5.8亿元，加快实施苏木乡镇中心幼儿园、义务教育学校标准化、薄弱学校改造、中等职业学校基础能力建设工程；支持赤峰学院打造区域人才培养高地和文化科技创新基地，加快交通职业技术学院高职示范校及综合实训中心建设，培育扶持赤峰工业职教集团健康发展。进一步改善城乡医疗卫生基础设施，开工建设市传染病医院综合病房楼，建成146个基层卫生医疗机构，打造蒙东区域医疗中心；加快建设全市卫生信息化平台和城乡居民健康档案数据库，建成市县乡村一体化公共卫生信息系统；推行城乡居民健康卡制度，实现全市范围内看病就诊一卡通。确保自治区第十三届运动会比赛场馆5月底全部建成并投入使用，全力承办一届隆重、节俭、安全、文明的体育盛会；加强群众体育设施建设，新建30个苏木乡镇健身广场，用两年时间实现苏木乡镇全覆盖。开工建设红山文化展示中心，加快推进红山文化遗址、辽代上京城和祖陵遗址的申遗工作；办好第九届红山文化节暨第三届中国印城文化节。抓好第三次经济普查工作。广泛开展社会主义核心价值观教育实践活动，扎实开展群众性精神文明创建活动，争创全国文明城市。继续做好人防、外事、侨务、档案、地震、气象、红十字等工作。

　　办好十件民生实事。在全面完成上级部署的惠民实事基础上，自加压力，挖掘潜力，集中财力再办一些普惠群众的实事好事。1.筹措资金4.2亿元，开工建设赤峰老年社区，使近1万名城乡老年人受益。2.面向贫困家庭和蒙古语授课高校毕业生，提供1900个基层公益性岗位。3.从2014年秋季起，实行蒙古语授课幼儿教育免费制度。4.筹措资金35.5亿元，建设廉租、公租住房1731套，实施老旧小区改造200万平方米，改造城市棚户区4623户、农村牧区危房2.15万户。5.筹措资金1.2亿元，在赤峰新区新建初中和小学各一所，蒙、汉语授课幼儿园各一所。6.筹措资金8亿元，新建通村油路800公里，新增通沥青水泥路嘎查村100个。7.筹措资金2.6亿元，实施饮水安全工程536处，解决30万人饮水问题。8.开展杠杆式金融扶贫富民工程，融资10亿元，使6万农牧民受益。9.开展政策性农业保险，按财政资金与群众自筹9:1的比例，筹措资金3.1亿元，承保面积1200万亩。10.筹措资金6.6亿元，为低收入农牧户每户供应一吨冬季取暖用煤。

　　（六）围绕城乡统筹发展，在加快新型城镇化建设方面有新作为，全面提升城乡一体化发展水平

　　加快百万人口区域中心城市建设。完成新一轮《赤峰市近期建设规划》、《陈营子组团控制性详规与核心区城市设计》以及《中心城区景观系统规划》等编制工作。理顺中心城市规划管理体制，强化规划集中统一管理。加大市政基础设施建设力度，科学利用城市地上、地下空间，实施供热、供气、停车、环卫、给排水及城市绿化、美化、亮化等项目37个，年内完成投资62.1亿元。推进治堵保畅工程，开工建设铁南大街，实施306国道城区段出口拓宽、西大桥重建、西站大街及公铁立交桥、天义桥、跨阴河桥梁等组团连接路工程，完成机场路建设，力争中环路新区段全线贯通；进一步渠化城区交通路口，优化信号灯设置，增开公交线路，积极推进中心城区周边50公里城乡公交一体化。进一步增加航线，加密航班，力争通航城市达到20个，进出港旅客突破90万人次。启动八家组团下洼子村、小新地组团新地村、临潢大街东段等棚户区改造工程，推动新老城区结合部明显改观。加强城市综合执法，提升城市管理水平。整合住建、公安、国土、电信等部门信息资源，搭建公共信息服务平台，提升城市管理智能化水平。

　　提高城镇承载能力。按照人口、资源、环境协调发展理念和中心城市标准建设县城、县城标准建设苏木乡镇的要求，科学论证人口、产业、水资源、土地、交通、环境等综合承载能力和容量，打造科学合理的市域城镇体系。从区位交通、资源禀赋、人文历史、群众意愿出发，更加注重乡土特色，更加注重现代文明元素融入，加快建设一批亮点纷呈的特色小城镇。做大做强县域经济，推进产城融合、城乡一体，强化就业、住房、消费、基本公共服务等生产生活要素保障，吸引广大农牧民向城镇集聚。启动实施旗县区城镇50个出入口综合治理工程。加快天义、大板两个次中心城市建设，推进宁城县撤县设市工作。大力扶持建筑业发展，增强市场竞争力。

　　建设美丽乡村。制定全市乡村发展规划，与城镇规划有效衔接，推进产业、设施、服务向农村牧区延伸拓展，提升城乡一体化发展水平。按照自治区用3年时间实现农村牧区基本公共服务十个全覆盖的部署，搞好规划，制定方案，明确工作任务，落实责任主体，抓紧启动实施；从实际出发，结合各旗县区新农村新牧区建设，适度集中地处偏僻、过于分散的行政村落和自然村庄，在城镇周边、园区及公路沿线建设一批设施完善、环境优美的农牧民新村。实施乡村清洁工程，在乡镇驻地村、乡域中心村、平川连片村、沿河沿路村等重点嘎查村集中开展庭院绿化、改水改厕、清理柴草、清除粪堆、清扫垃圾等专项治理活动，努力实现村庄美化、路院硬化、河渠净化，让农村牧区成为安居乐业的美丽家园。

　　（七）围绕平安赤峰建设，在创新社会治理方面有新作为，共建和谐幸福家园

　　支持社会组织发展，加强行业协会、商会、社会服务、公益慈善等社会组织建设，做好孵化培育、等级评估和授权工作，确保政府职能转得出、接得住、管得好。加快社会组织去行政化，逐步实现行业协会、学会、研究会与党政机关、事业单位彻底分开。加强行业自律与诚信建设，探索建立黑名单制度。推进嘎查村级事务契约化、社区化管理，提高群众自我管理、自我发展能力。整合苏木乡镇、城乡社区的社会管理资源，推进网格化管理、精细化服务模式，提升社区管理服务水平。加强社工队伍建设，积极发展各类志愿服务组织。

　　大力化解社会矛盾。积极推广枫桥经验，切实发挥基层组织预防化解社会矛盾的重要作用，努力做到小事不出嘎查村、大事不出苏木乡镇、矛盾不上交。深入推进人民调解、行政调解和司法调解相互衔接的大调解体系建设。严格落实信访工作领导责任制，坚持领导干部接访、下访、包案办理制度，健全疑难复杂信访事项公开听证制度，有针对性地解决群众反映的信访突出问题。按照事要解决原则，建立属地管理、分级负责制，依法及时就地解决群众合理诉求，化解信访积案。拓宽信访渠道，大力推进网上受理办理信访制度。建立涉法涉诉信访依法终结制度。全面落实社会稳定风险评估机制，突出抓好前置准入、决策评估和事后问责三个环节，推动社会矛盾防控和化解工作规范化、常态化。

　　切实加强安全监管。监督企业加大安全投入、履行主体责任，最大限度地消除安全隐患。严格落实安全生产一岗双责制，完善联合执法工作机制，按照谁主管、谁负责，谁审批、谁负责原则，加强矿山、化工、建筑、交通、消防等高危行业和学校、医院、车站、商场等重点领域的安全监管，坚决遏制重特大事故发生。完善食品药品安全监管体制。加快农畜产品质量安全检验检测和追溯体系建设，强化生产源头和产销全程监管，建立严格的安全生产规范，确保人民群众舌尖上的安全。严格执行安全生产一票否决制，依法严肃查处各类安全事故。

　　扎实推进平安赤峰建设。完善劳动标准体系和劳动关系协调机制，严厉查处克扣拖欠工资等违法行为。依法推进社区矫正和法律援助工作，深入实施六五普法规划。加快应急管理基础建设，完善灾害事故和公共安全事件预警、应急处置体系。加强公共安全宣传教育和应急演练，提高广大群众自我保护意识和应对突发事件能力。加强城乡社区警务、政法综治组织和群防群治等基层基础建设，增强公共安全和社会治安保障能力。完善立体化社会治安防控体系，始终保持对各类违法犯罪活动的高压态势，持续提升人民群众的安全感和满意度。全面贯彻落实党的民族宗教政策，依法管理民族宗教事务。进一步增进军政军民团结，支持军队和国防建设，力争实现全国双拥模范城八连冠。

　　（八）围绕解决四风问题，在推进政府自身建设方面有新作为，树立创新务实、廉洁高效、敢于担当、风清气正的良好形象

　　面对经济形势的新变化和全面深化改革的新要求，进一步解放思想、创新实践，以自我革新的勇气和胸怀，跳出条条框框，全方位更新思想观念、转变思维方式、振奋精神状态。自觉强化功成不必在我的境界，不贪一时之功，不图一时之名，顾大局，识大体，脚踏实地，甘于奉献。牢固树立危机意识，从解决当前制约发展最迫切、最现实的问题入手，紧盯薄弱环节，敢涉险滩，善谋对策，勇破难题，努力掌握科学发展主动权。切实增强一日无为、三日不安的紧迫感，定下的事雷厉风行，看准的事一抓到底。发扬钉钉子精神，稳扎稳打，实干苦干，步步推进，确保各项工作全面落实到位。完善重大事项行政决策机制，注重调查研究，广泛听取群众意见，不断提高科学决策和民主决策水平。全面优化政务环境，宁可让机关伤筋动骨，不让群众和企业费力伤神。按照中央和自治区统一部署，扎实开展第二批党的群众路线教育实践活动，集中解决四风问题，着力整治庸懒散、奢私贪、蛮横硬等不良现象，严厉查处吃拿卡要、以权谋私等违法违纪行为。继续取消和下放一批行政审批事项，确保不打折扣、不搞变通。加强各级政务服务中心规范化建设，进一步优化审批流程，推行首问负责、联审联批、限时办结制度。加快电子政务建设，在公安、社保、房管、工商、税务、医保、公积金等重点领域实行网上预约、在线办理。全面清理市县乡政府利用红头文件设定的管理、收费、罚款项目，让企业和群众切实感受到简政放权带来的实惠和便利。科学设置考核指标，更加注重对资源消耗、环境保护、安全生产、扶贫开发、政府债务等方面的绩效考核。严格遵守中央关于党政机关厉行节约反对浪费、国内公务接待、差旅费管理等各项规定，进一步压缩三公经费和一般性支出，不新建政府性楼堂馆所、不增加财政供养人员，全面清理超标准办公用房和违规公务用车。深入开展廉政建设和反腐败斗争，强化对行政权力的制约监督，加大行政问责力度，加强对重点领域、重点项目、重点资金的审计监督，把权力关进制度的笼子里。

　　各位代表，我们深知，政府的一切权力都是人民给的，政府工作必须置于人民的监督之下。我们一定自觉接受人大及其常委会法律监督和工作监督，自觉接受政协民主监督，自觉接受人民群众监督和舆论监督，虚心听取各民主党派、工商联、离退休老干部和社会各界人士的建议和意见，主动加强与工会、妇联、共青团等人民团体联系，不断改进政府工作，认真履行好政府职责，不辜负全市人民的信任和期望。

　　各位代表，团结凝聚力量，奋斗铸就辉煌。我们坚信，任何困难都改变不了赤峰人民实现美好梦想的不懈追求，任何挑战都阻挡不了赤峰大地推进科学发展的坚实脚步。让我们深入贯彻落实党的十八大、十八届二中、三中全会精神和习近平总书记视察内蒙古时的重要讲话精神，守望相助，团结拼搏，快马加鞭，为全面推进科学发展、富民强市伟大进程而努力奋斗！
